# Democratizing Artificial Intelligence Imaging Analysis With Automated Machine Learning: Tutorial

**DOI:** 10.2196/49949

**Published:** 2023-10-12

**Authors:** Arun James Thirunavukarasu, Kabilan Elangovan, Laura Gutierrez, Yong Li, Iris Tan, Pearse A Keane, Edward Korot, Daniel Shu Wei Ting

**Affiliations:** 1 University of Cambridge School of Clinical Medicine Cambridge United Kingdom; 2 Artificial Intelligence and Digital Innovation Research Group Singapore Eye Research Institute Singapore Singapore; 3 Moorfields Eye Hospital NHS Foundation Trust London United Kingdom; 4 Byers Eye Institute Stanford University Palo Alto, CA United States; 5 Retina Specialists of Michigan Grand Rapids, MI United States; 6 Singapore National Eye Centre Singapore Singapore

**Keywords:** machine learning, automated machine learning, autoML, artificial intelligence, democratization, autonomous AI, imaging, image analysis, automation, AI engineering

## Abstract

Deep learning–based clinical imaging analysis underlies diagnostic artificial intelligence (AI) models, which can match or even exceed the performance of clinical experts, having the potential to revolutionize clinical practice. A wide variety of automated machine learning (autoML) platforms lower the technical barrier to entry to deep learning, extending AI capabilities to clinicians with limited technical expertise, and even autonomous foundation models such as multimodal large language models. Here, we provide a technical overview of autoML with descriptions of how autoML may be applied in education, research, and clinical practice. Each stage of the process of conducting an autoML project is outlined, with an emphasis on ethical and technical best practices. Specifically, data acquisition, data partitioning, model training, model validation, analysis, and model deployment are considered. The strengths and limitations of available code-free, code-minimal, and code-intensive autoML platforms are considered. AutoML has great potential to democratize AI in medicine, improving AI literacy by enabling “hands-on” education. AutoML may serve as a useful adjunct in research by facilitating rapid testing and benchmarking before significant computational resources are committed. AutoML may also be applied in clinical contexts, provided regulatory requirements are met. The abstraction by autoML of arduous aspects of AI engineering promotes prioritization of data set curation, supporting the transition from conventional model-driven approaches to data-centric development. To fulfill its potential, clinicians must be educated on how to apply these technologies ethically, rigorously, and effectively; this tutorial represents a comprehensive summary of relevant considerations.

## Introduction

Automated machine learning (autoML) is the product of attempts to broaden artificial intelligence (AI) engineering capability beyond those with technical and computational expertise [1]. Machine learning (ML) is a form of AI that describes the computational process of leveraging data to improve performance in a defined task, thereby developing sophisticated models without explicit programming. More recently, deep learning (DL) has emerged as a powerful form of ML capable of interpreting unstructured data, such as images, language, and speech [2,3]. In DL, layers of representation are developed that iteratively manipulate input data until useful features emerge, permitting the processing of highly complicated data sets. These layers are composed of tuned artificial neurons; computationally encoded mathematical functions that together comprise a deep neural network. Results across medicine have been impressive, with the production of many models with expert or beyond-expert accuracy, sensitivity, and specificity [4]. AutoML acts to extend automation even further through various aspects of algorithm development, including hyperparameter optimization and neural architecture search [1].

**Figure 1:**
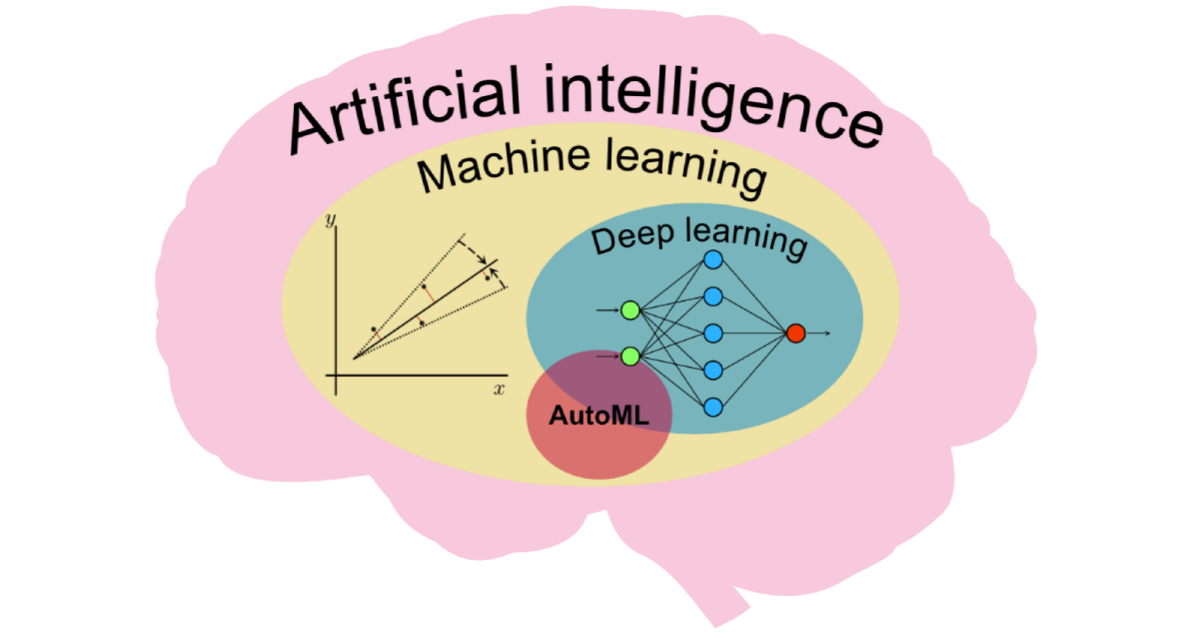
Relationship between autoML, deep learning, machine learning, and artificial intelligence. autoML: automated machine learning.

Many autoML platforms have been developed in industry and academia, with recent innovation producing platforms capable of DL, compatible with unstructured input data such as medical images (Figure 1) [5,6]. These platforms, with different requirements, capabilities, and limitations, may be categorized based on the spectrum of user coding requirements (Table 1). To capitalize on the potential of AI, more users must be able to harness DL and other ML techniques, leveraging the health care data that continues to be accrued at an accelerating rate [7]. This reduction in the requirement for expertise and computational requirements constitutes the “democratization” of AI technology [5,8]. Democratization refers to the broadening of access to technology conferred by reduced technical and hardware requirements.

When effectively deployed, DL has the potential to improve patient safety, quality of health care, and cost-effectiveness [9-11]. These improvements are based on the accuracy, speed, and reproducibility of DL algorithms, which can exceed that of humans with extensive training [9,10]. Accurate and reliable computational models may complement skilled human assessments as a part of novel systems with equivalent or superior performance to conventional practice with the additional benefit of being less expensive [11]. Successful projects benefit from interdisciplinary collaboration, with clinical and technical expertise brought to bear [12]. Clinicians, computer scientists, and data scientists work together, and their time and resources are scarce [13-15]. Collaboration also introduces complications regarding communication, particularly where individuals’ expertise differs, and regarding sharing patient data for which privacy is closely governed [16]. Increasing the accessibility of high-performance DL for clinicians with autoML may ameliorate these issues [6]. Through the democratization of DL, a greater number of AI-literate clinicians can contribute to the research, implementation, and governance of these systems [17]. Moreover, emerging AI models with the capability to leverage application programming interfaces as tools could facilitate AI building itself at a rapidly accelerating rate; early examples include GPT-4, PaLM 2, and LLaMA 2 [18].

Below, we show how to use autoML for medical image analysis for clinicians and other interested allied health care professionals, with step-by-step illustrations of workflow, important considerations, and requirements. The strengths and weaknesses of code-free, code-minimal, and code-intensive platforms are discussed, in addition to the capabilities and limitations of each platform. The potential use cases of autoML in education, primary research, and clinical practice are outlined. The technical and ethical best practices are emphasized throughout to encourage maintained or even improved standards of development and reporting, as a broader subset of clinicians gain access to AI and as developers look to adopt a data-centric approach to constructing models [19].

**Table 1:** Summary of autoML platforms facilitating deep learning for images, with an appraisal of accessibility and portability.

AutoML^a^ platform	Accessibility				Portability	
	Cost	Code requirement	Computing location	Exportability		Explainability
Amazon recognition	Chargeable	None	Cloud	No		No
Apple create ML^b^	Free on specific devices	None	Local	To Apple devices		No
Auto-PyTorch	Free	Coding required	Local	Yes		No
AutoGluon	Free	Coding required	Local	Yes		Yes
AutoGOAL	Free	Coding required	Local	Yes		No
AutoKeras	Free	Coding required	Local	Yes		No
Baidu EasyDL	Chargeable	None	Local or cloud	To edge devices		No
Clarifai	Free (chargeable features)	None	Cloud	To edge devices		No
Google Cloud AutoML Vision	Chargeable	None	Cloud	To edge devices		Yes
Huawei ExeML	Chargeable	None	Cloud	No		No
H2O.ai Hydrogen torch	Chargeable	None	Local or cloud	Yes		Yes
H2O R/Python packages	Free	Coding required	Local	Yes		Yes
H2O.ai Driverless Al	Chargeable	None	Local or cloud	Yes		Yes
KNIME	Free (chargeable features)	None	Local	Yes		No
MATLAB	Chargeable	Coding required	Local	Yes		No
MedicMind	Free (chargeable features)	None	Cloud	Yes		Yes
Microsoft Azure AutoML	Chargeable	None	Cloud	To edge devices		No
Neuro-T	Chargeable	None	Local	Yes		No
Sony prediction one	Chargeable	None	Local or cloud	Yes		Yes

^a^AutoML: automated machine learning.

^b^ML: machine learning.

### Technical Overview

**Figure 2:**
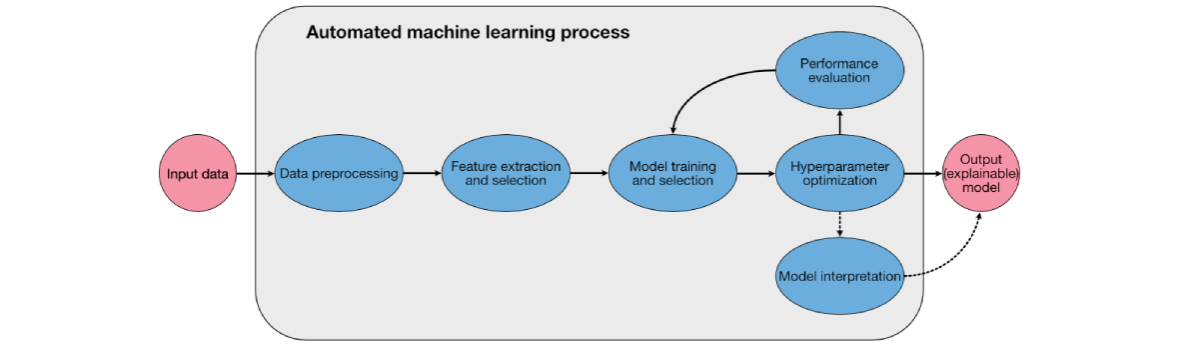
A conceptual diagram of automated machine learning, which may generate a predictive model from input data in the form of medical images. Data preprocessing entails the processing of inputs to augment and simplify the data, and "clean" data into a compatible format. Feature extraction involves the identification of the elements of the input data, which provide the most discriminative power. Model selection, training, and optimization summarize the process of training a myriad of potential deep learning architectures, selecting the best-performing architecture, and optimizing hyperparameters such as time to train or the number of iterations using training data. To judge which model is optimal, and to report its effectiveness, performance evaluation is required. Some automated machine learning platforms facilitate interpretation, allowing the deduction of how decisions are reached.

In general, autoML technology executes part or all of the ML engineering process without users’ input (Figure 2). Without autoML, these tasks require skilled data or computer scientists. Through a process of trial and error, informed by prior experience, these experts attempt to find an optimal neural network structure and hyperparameters to solve clinical problems, such as disease diagnosis, treatment planning, or prognosis prediction. AutoML has been applied primarily to classification tasks thus far, where an algorithm seeks to correctly identify (“classify”) images exhibiting one of a defined set of potential conditions or diseases (“classes”) [5,6]. A wide variety of ML algorithms such as k nearest neighbors, support vector machine, random forest, neural network, naive Bayes, and logistic regression exist for classification, from which an autoML platform may select depending on comparative performance [20]. This is an example of supervised learning, as input data must be labeled by defined classes. In contrast, autoML for unsupervised and reinforcement learning is relatively nascent [21,22].

To achieve performance comparable to bespoke ML models trained by computer scientists, autoML platforms use a variety of methods and optimization techniques including Bayesian optimization, random search, grid search, evolutionary-based neural architecture selection, and meta-learning [23]. An optimal model may then be outputted for internal or external validation, interpretation, and deployment. Many platforms, with various accessibility, technical features, and portability, have been developed in academia and industry. When deciding which platform to use, researchers and clinicians should consider their capabilities, requirements, and aims.

### Workflow

**Figure 3:**
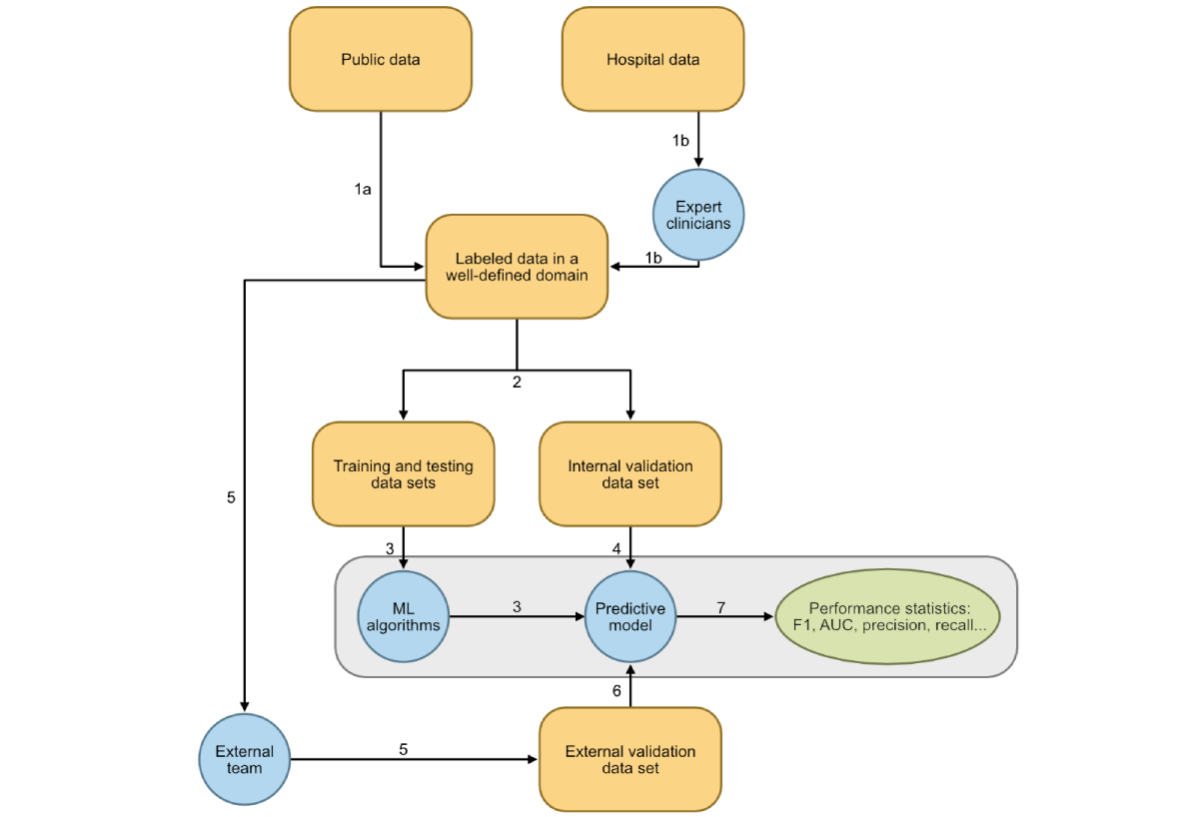
The process of developing a deep learning model with automated ML. Minimally expected processes facilitated by an automated ML platform are within the gray-shaded region. However, platforms variably assist with other processes. 1A: public data set curation; 1B: private data set curation; 2: partitioning; 3: training; 4: internal validation; 5: curation of independent data set; 6: external validation; 7: performance evaluation and results presentation. AUC: area under the curve; ML: machine learning.

The process of applying DL to medical image analysis generally involves gathering high-quality data, training a model, and evaluating its performance; the reporting of these processes has been standardized [24]. The process of applying autoML is comparable, despite being less technically demanding, and still relies on careful selection and labeling of representative data sets for the designated use case (Figure 3). These data-driven principles are applicable across different imaging modalities, including X-ray, ultrasound, computed tomography, magnetic resonance imaging, optical coherence tomography, fundus photography, and angiography [25]. The algorithms can be trained very quickly using autoML with expert or even supraexpert ability to classify medical images [6].

### Data Set Curation

#### Data Management

For trained algorithms to be appropriate for clinical deployment, it is helpful to use data in the same format as in routine clinical practice. This may be complicated where different formats exist within a single imaging modality, such as from different machines or scanning protocols; an ML model may struggle to classify images due to insufficient salient clinical features relative to structural differences in diverse input. Converting images to a common format may involve manual formatting, down-sampling resolution, or labeling classes with folders or file names. Larger data sets are often assumed to be less susceptible to weaknesses but are not a solution in themselves [26]. In some cases, larger data sets lead to worse performance in a classification task, as seen in published studies attempting to distinguish cognitively normal, mild cognitive impairment, and patients with Alzheimer disease [26]. However, the sample size must be sufficient to represent the general population to which algorithms may be applied, and requirements are more stringent where disease features vary widely or where differences between patients are subtle.

#### Publicly Available Data (Step 1A)

Many data sets curated to support ML research exist, covering a wide variety of imaging modalities and clinical diagnoses [27,28]. These permit researchers to download and use data to train and test ML models including autoML, but attention to specific permissions is required to avoid breaching privacy or copyright regulations. Data sets often make specific requests of researchers using their files, such as citing a source study in any resultant published work. Furthermore, they often have licensing limits for the development of commercial algorithms derived from the posted data. Where privacy permissions are restricted, robust data security is essential to ensure patient information is not shared with unauthorized parties.

Public data sets are often prelabeled according to a condition of interest. For researchers, it is essential to determine the source of labels and adjudge whether the labeling process is suitably rigorous to use for their specific project, and if the “ground truth” (data set labels that are assumed to be true during training) is sufficiently accurate. If quality standards are not met, such as if labeling was conducted by insufficiently skilled clinicians, if there was no arbitration or adjudication process, or if mistakes are apparent, researchers need to arrange relabeling or correct the wrong labels themselves. By improving the quality of input data, algorithm performance may be significantly stronger, with fewer errors expected [29].

#### Private Data (Step 1B)

Private data sets may be curated by researchers as part of an autoML project. This requires institutional review board ethical approval, as well as informed consent from patients to use their data in an explicitly defined research context. As discussed above, data security is of paramount importance to ensure consent is not breached regarding the persons given access to patients’ data. Sharing data with commercial autoML platforms may be prohibited, necessitating the use of local autoML platforms, such as H2O, Apple CreateML, or autoKeras. Deidentification of data may be a requirement to obtain research ethical approval, retaining only essential elements to facilitate labeling and classification [30].

Labeling should be undertaken by the research team. While certain autoML platforms assist with labeling through active learning, these are often cloud-based services that may require additional institutional approval before use. Labeling may be based on incontrovertible pathologic, genomic, or clinical outcome ground truth or a less accurate approximation based on annotations by expert clinicians, preferably conducted prospectively as clinicians may thereby incorporate contemporaneous clinical and laboratory data into their decision-making. Rigorous arbitration and adjudication are required to minimize labeling errors, which may otherwise significantly impair model performance [30,31].

#### Partitioning (Step 2)

Data partitioning is a critical preliminary step when building any ML model in order to evaluate model performance fairly on an unseen, representative data set independent from the images used to train the model. Data must be split into training and testing partitions, and there is a wide range of partitioning algorithms with different strengths and limitations summarized as round-robin, hash, range, and random schemes [32]. As a rule of thumb, 80% of available data may be used for training, with 20% used for testing (internal validation). Most autoML platforms facilitate the upload of separate partitions corresponding to training and testing; otherwise, the platforms themselves split data accordingly. Automatic partitioning with obtuse algorithms results in training and testing data sets that may not be known to the user, precluding the establishment of representativeness of these data sets. Therefore, it is best practice to manually partition data sets with patient-level splits, using reproducible and documented methods.

#### Training and Internal Validation (Steps 3 and 4)

Reliance on a single data set (especially if small) may result in “overfitting”—where algorithms learn features specific to the images in the training data set only [33]. While performance may be exceptional on the training data set, it is weaker when algorithms are applied to unseen data. To avoid this trap, a small subset of data must be reserved for validation, which acts as a means of observing model performance at each training iteration to guide the process and adjudge when algorithms have been optimally entrained. Separation is key, as algorithms are expected to perform accurately with images previously “seen”; the training data set has features identical to those “learned” by a model to be associated with classification labels. By using a data set entirely separate from the training process, a fairer evaluation of model performance may be obtained and summarized with an array of statistical metrics (discussed in step 7). In practice, ML models improve accuracy on this internal validation data set as the primary indicator of successful training: the final algorithm corresponds to that which performs best on the internal validation data set, where classification accuracy is maximized without overfitting to the training data. Many autoML platforms use cross-validation, where multiple partitioning processes are applied to generate separate testing and training data sets. In this case, model selection is based on optimal performance across iterations over all the partitioned data sets, further reducing the risk of overfitting [33].

#### Curation of an Independent Data Set (Step 5)

To externally validate a predictive model, data entirely separated from the training and testing process must be used. External validation demonstrates true generalizability if performance is acceptable with diverse data sets, representative of future cohorts where models may be used with a range of idiosyncratic differences inherent in obtaining images in different clinical environments. While the curation process is identical to that described in steps 1A and 1B, there exists an additional option to collect data prospectively to facilitate a robust analysis of model performance in clinical conditions, with a lower risk of bias [34]. This entails obtaining ethical approval and may require patient consent, as well as the time and clinical training required to collect suitable data. Conducting a prospective pragmatic trial would represent the strongest form of primary evidence for justifying deployment in clinical settings in the future.

#### External Validation (Step 6)

Using an independent data set on the same model is important to demonstrate generalizability beyond the restricted data used to initially train and test the autoML algorithm. This is most conveniently executed through batch prediction of an external validation data set, but platforms may restrict processing to single images or prohibit the export or deployment of a model without extra costs [6]. While external validation is preferably undertaken by a separate research team to avoid a potential source of bias, initial validation may be done by the same team to improve the veracity of their performance claims [35,36]. If open-source data sets are used, performance with as many data sets as possible should be reported to avoid selection bias resulting from cherry-picking of data where performance is higher; this may be due to the external validation data set being more similar to the training and internal validation data set.

#### Performance Evaluation and Results Interpretation (Step 7)

Model performance metrics and visualizations are the most important features to users in terms of developing trust in an autoML platform [37]. Many metrics are used in ML research; some are a function of prevalence such as accuracy, area under the precision-recall curve (PRC), and F1 score, whereas others are a function of the model threshold, such as accuracy and F1 score [38]. Threshold refers to the cutoff point of prediction probability above which the model gives one output or another, governing sensitivity and specificity. Many autoML platforms provide just a few performance metrics, in part due to displaying results from an “optimal” model operating at a single threshold, which prohibits calculation of metrics such as area under the precision-recall curve or area under the receiver operating characteristic curve (ROC), and PRC and ROC plots cannot be produced without implementing the model at a range of thresholds. With only a snapshot of performance statistics at one model threshold, it is possible that apparent performance is inflated by condition prevalence or model hypersensitivity. In addition, threshold customizability increases the likelihood of a model being clinically useful. While a particular performance metric may be maximized at 1 threshold, there may be a requirement for tuning, such as to optimize the sensitivity or specificity. Providing more metrics may allow fairer comparison to alternative computational techniques and expert clinician performance, and confusion matrices are an essential tool to judge the use of a model’s performance. Certain platforms do provide the customizability to generate PRC and ROC plots, but these often have a greater requirement for coding.

Explainability is an ML research priority due to concerns over delegating responsibility to “black box” models. By understanding how models make successful predictions, the potential risks of delegating decision-making to systems with occult biases are avoided [39]. Clinicians and patients may have more confidence in the so-called explainable AI. The availability of transparency features on autoML platforms is recognized as a key aspect of users’ trust and understanding when using these tools [37]. Some platforms have inbuilt explainability features; examples include H2O.ai Driverless AI and Google Cloud AutoML, which provide Grad-CAM and XRAI-derived saliency maps depicting which parts of an image contributed to classification [40]. However, these tools leave an “interpretability gap,” which can lead to misleading conclusions [41]. Further explainable AI innovation is required, but this is complicated with platforms that do not facilitate model export and deployment on new batches of data, as with external validation. New tools are being developed to facilitate the interpretation of ML and even autoML models less amenable to export; examples include the What-If Tool which facilitates counterfactual analysis of model performance and individual classification decisions as input data are altered [42].

#### Capabilities and Limitations: Platform Comparison

The wide variety of autoML platforms offers different capabilities and limitations. In general, platforms may be discussed in terms of their requirement for coding ability, with code-free, code-minimal, and code-intensive examples (Table 1). Platforms may also be parsed by the location of data and processing as either cloud-based or local. Cloud-based solutions may be more secure than local solutions due to industry-standard encryption and International Organization for Standardization compliance audits but require explicit ethical approval to be used with sensitive patient data. Without ethical approval, using cloud-based autoML is limited to open-source data sets, which are now abundant but often lacking in terms of quality labeling and representative populations [27,28]. While local platforms may be preferred for their tendency not to require payment for access, hardware requirements may be prohibitive and security protocols must be sufficiently robust, limiting their role in democratizing AI.

Technical features correspond to the development process outlined in Figure 2. The so-called “end-to-end” platforms may be defined as those that automate all these processes; all users are required to do is input labeled data. Most platforms equipped for DL have end-to-end functionality, operating without a requirement for user input. Platforms differ in their permissiveness of model export for further validation, explainability analysis, and potential deployment. In general, local platforms always facilitate the export of models amenable to explainability analysis, external validation, and deployment at scale. Cloud-based platforms are generally more of a “black box,” offering no details as to the model architecture, but some enable model export and batch prediction to facilitate external validation and explainability analysis—often in return for a fee.

### Use Cases of AutoML

#### Medical Education

AutoML for medical image analysis can be an educational tool for clinicians and medical students. By lowering the requirement for coding expertise or GPU access, autoML permits more learners to explore ideas practically rather than merely discussing them in theory. Learners can actively produce and modify models to demonstrate the importance of data set quality, validation, and explainability for themselves [43]. This may also provide learners with the intuition of an ML developer sooner—conferring greater practical expertise than a mere understanding of theory alone when approaching new problems. As autoML promotes interaction with data over coding, further learning through institutional courses or individual initiatives is required to develop the necessary ML expertise to engineer bespoke, fully customizable models. However, focusing on data may best prepare clinicians for future trends in ML development and promote mechanical understanding of algorithms rather than learning tricks to maximize performance.

A transition from model-driven to data-driven techniques—incubated and facilitated by autoML—has been discussed as a means of accelerating development, as arduous engineering is bypassed. This complements the recent drive to inculcate “data-centric AI” (spearheaded by Andrew Ng), where data set curation is focused on rather than optimizing code. As the supply of high-quality data is more often the limiting factor in development than code or model infrastructure, future innovation is likely to focus on the generation and aggregation of training data [19]. The approach is a nascent paradigm in medicine, although promising results have begun to emerge.

#### Medical Research

Another main use case for autoML is medical image-based primary research, including pilot studies and larger-scale projects. AutoML pilot studies, with relatively low costs in terms of time and money, may be used to gauge whether a research question can be solved with AI and ML techniques. AutoML enables clinicians to perform initial proof of concept studies with private and well-labeled data, generating initial outcomes without a requirement for collaboration with computational experts, enabling optimization of research resource allocation. For example, autoML may help determine whether a certain sample size or quality level of images is useful and practical for developing classification models. This is an alternative to haphazard trial and error, which wastes expertise, time, and resources as infeasibility is determined at a later step. External technical collaborators may be approached with promising interim results generated by autoML, increasing the likelihood of a proposed project succeeding. Applications for research funding may be strengthened by promising pilot study results generated with autoML.

AutoML may also be used independently in primary research. However, to apply autoML in medical image analysis, there are rigorous academic requirements, including standardization of benchmarks, ensuring reproducibility in analysis and the interpretability of outcomes, and following guidelines for research and reporting, such as for Developmental and Exploratory Clinical Investigations of Decision Support Systems Driven by Artificial Intelligence (DECIDE-AI) [24]. The technical limitations of certain autoML platforms make adherence to these standards more challenging, but useful results have been and will continue to be produced with autoML technology.

#### Clinical Deployment

As with all AI research in health care, models are hoped to improve patient care in real-world settings [44]. As studies have demonstrated the performance of models generated by autoML to be comparable or even higher than those generated with conventional AI techniques, there is a basis for exploring the clinical deployment of autoML. Although autoML often restricts explainability and interpretability—discussed above—many Food and Drug Administration-approved models have no explainability [45]. However, to be deployed in direct clinical care, autoML models must meet the same regulatory standards as conventionally developed AI applications. These standards are evolving around the world but involve extensive evaluation and validation and are often expensive application processes to regulatory bodies. For regulatory clearance, emphasis is placed on the intended clinical use; this must be defined precisely to ensure deployment as desired is permissible if an application is accepted. Requirements include demonstration of data traceability and quality, so careful documentation is required to preserve the source of data and ground truth. Model versions must be documented with the data used at each step; only the version accepted by a regulatory body may be deployed. Additional considerations include governance systems, adherence to software development cycle requirements and International Organization for Standardization regulations, integration into clinical workflow, and cybersecurity.

### Conclusions

AutoML is an exciting innovation that reduces the barrier to entry for AI development, including DL for medical image classification. With the democratization of AI, it is hoped that the quality and acceptance of AI innovations for patient diagnosis, management, and prognosis will improve, accelerating computational innovation in clinical practice. Specifically, empowering nonspecialists to harness DL technology may enable clinician-driven AI, allowing experts with knowledge of domain-specific pain points to take a more active role in the development of applicable, effective, and useful new tools. The technical limitations of autoML are reducing as corporate and academic developers continue to improve available platforms, although conventional techniques have an edge in terms of capability, customizability, and explainability. This currently limits the potential of autoML, particularly in younger, developing subfields such as multimodal AI and autonomous foundation models [46,47]. Nevertheless, autoML represents an excellent tool for interested clinicians to develop DL skills, conduct pilot studies and other research, and produce models to improve clinical practice.

## Abbreviations

AI: artificial intelligence
autoML: automated machine learning
DECIDE-AI: Developmental and Exploratory Clinical Investigations of Decision Support Systems Driven by Artificial Intelligence
DL: deep learning
ML: machine learning
PRC: precision-recall curve
ROC: receiver operating characteristic

## Data Availability

All data generated or analyzed for the work presented here are included in this published article.
